# Oxytocin Attenuates the Stress-Induced Reinstatement of Alcohol-Seeking in Male Rats: Role of the Central Amygdala

**DOI:** 10.3390/biomedicines9121919

**Published:** 2021-12-15

**Authors:** Hannah S. Ballas, Samantha M. Wilfur, Nicole A. Freker, Kah-Chung Leong

**Affiliations:** Department of Psychology, Trinity University, San Antonio, TX 78212, USA; hballas@trinity.edu (H.S.B.); swilfur@trinity.edu (S.M.W.); nfreker@trinity.edu (N.A.F.)

**Keywords:** oxytocin, stress, yohimbine, alcohol, reinstatement, central amygdala

## Abstract

Factors such as stress and anxiety often contribute to alcohol-dependent behavior and can trigger a relapse of alcohol addiction and use. Therefore, it is important to investigate potential pharmacological interventions that may alleviate the influence of stress on addiction-related behaviors. Previous studies have demonstrated that the neuropeptide oxytocin has promising anxiolytic potential in mammals and may offer a pharmacological target to diminish the emotional impact on reinstatement of alcohol-seeking. The purpose of the present study was to investigate the effect of oxytocin on stress-induced alcohol relapse and identify a neural structure mediating this effect through the use of an ethanol self-administration and yohimbine-induced reinstatement paradigm. While yohimbine administration resulted in the reinstatement of ethanol-seeking behavior, the concurrent administration of yohimbine and oxytocin attenuated this effect, suggesting that oxytocin may disrupt stress-induced ethanol-seeking behavior. The central amygdala (CeA) is a structure that drives emotional responses and robustly expresses oxytocin receptors. Intra-CeA oxytocin similarly attenuated the yohimbine-induced reinstatement of ethanol-seeking behavior. These results demonstrate that oxytocin has the potential to attenuate stress-induced relapse into ethanol-seeking behavior, and that this mechanism occurs specifically within the central amygdala.

## 1. Introduction

Alcohol use disorder (AUD) is an urgent public health concern within the United States, with lifetime prevalence rates exceeding 29% among Americans [1]. Recovering addicts often cite negative emotions, such as stress and anxiety, as major contributing factors that facilitate the initiation and relapse of alcohol use after a period of abstinence [2]. Despite the well-documented effects of stress on alcohol-related behaviors, there are few clinically-relevant pharmacotherapies available to alleviate the negative impact of stress on these maladaptive processes.

Oxytocin (OXT) is a well-characterized neuroendocrine hormone produced within the hypothalamus, and has recently gained interest for its robust anxiolytic effects [3,4,5]. OXT administration has been found to reduce anxiety-related behaviors and the physiological consequences of stress and anxiety in rodents. Similarly, higher levels of oxytocin were correlated with lower levels of anxiety and the acute intranasal administration of OXT reduced anxiety symptoms in humans [6,7]. Animal studies involving rats demonstrated that the administration of OXT diminished behavioral responses in situations involving social stress [8] and unpredictable environmental threats [9]. OXT administration has also been shown to decrease stress-induced corticosterone release and anxiety-related behaviors in rats [10], and several studies have demonstrated a strong anxiolytic effect of OXT administration in rodents [5,11] and humans [3]. Early evidence also suggests that OXT effectively attenuates anxiety-induced reward-seeking behaviors [12,13], although the specific neural mechanism underlying this effect has not been fully characterized.

Magnocellular neurons of the paraventricular nucleus send extensive oxytocin projections to the central amygdala (CeA), which has been found to express oxytocin receptors (OXTRs) [14,15]. Several studies have demonstrated that oxytocin’s anxiolytic effect may be mediated by mechanisms within the CeA, with the intra-CeA administration of OXT resulting in diminished fear responses [16] and endogenous OXT into the CeA suppressing fear expression [17]. The CeA has also been found to be a key structure underlying stress-induced drug-seeking behavior, with several studies implicating the CeA as a key structure in driving stress-induced reinstatement for several drugs of abuse including cocaine [18,19], ethanol [20], and nicotine [21].

The operant self-administration and reinstatement model of addiction [22,23] is a useful translational animal model for addiction research, particularly for stress-induced relapse behaviors. In this paradigm, rats are trained to perform an instrumental/operant response (e.g., lever press) for the contingent self-administration of a drug (i.e., primary reinforcer). Rats are subsequently relieved of this operant response. Drug-seeking behavior can then be re-established following stress exposure. Here, we employed an ethanol self-administration and reinstatement paradigm, in which the reinstatement of ethanol-seeking behavior is driven by acute exposure to the anxiogenic drug yohimbine (YOH). In the present study, we established that systemically administered OXT may provide a viable pharmacological intervention to attenuate the effect of YOH-induced alcohol-seeking behavior. We also determined that this attenuating effect of OXT is driven by processes within the CeA, as the intra-CeA infusion of OXT similarly attenuated alcohol-seeking behavior driven by YOH administration.

## 2. Materials and Methods

### 2.1. Animals

Adult male (maintained at 275–300 g throughout study) Sprague-Dawley rats (Charles River Laboratories, *n* = 20) were used in this study. The rats were single-housed on a reverse 12:12 light-dark cycle in a set-temperature and humidity-controlled vivarium. During the experiment, the animals were food-restricted to 20 g of chow daily in order to maintain a weight compatible with surgical procedures. The animals were water-restricted for 4 h prior to behavioral training during the initial phases of self-administration until operant responding was established, after which they received water ad libitum. All the procedures were approved by the Institutional Animal Care and Use Committee (IACUC; 082621-KCL) of Trinity University.

### 2.2. Apparatus

All the self-administration, extinction, and reinstatement procedures were carried out in a metallic operant chamber (30.48 cm × 25.40 cm × 30.48 cm) containing a metal grid floor and an overhead activity counter (Coulbourn Instruments, Holliston, MA, USA). Each chamber contained a house light, two retractable levers (one active, one inactive) with cue lights above each lever, and an access-controlled optical lickometer behind a guillotine door. The lickometer was connected to a standard drinking tube and contained photocell sensors capable of measuring the number of licks each animal produces on the drinking tubes. The chamber was located inside a sound-attenuating cabinet with an ambient fan. All efforts were made to minimize any external smells or sounds and the subjects were only run during the dark phase of their 12 h opposite light–dark cycle.

### 2.3. Drugs

The oxytocin (Cell Sciences, Newburyport, MA, USA) was dissolved in 0.9% NaCl saline and administered intraperitoneally (i.p.; 1 mg/kg at a volume of 1 mL/kg) or infused intra-CeA (0.5 µg) at a volume of 0.5 µL. This volume was selected based on previously published studies showing localized pharmacological effects within the rat CeA [18,24]. The yohimbine (Acros Organics, Carlsbad, CA, USA) was dissolved in water and administered intraperitoneally (i.p.; 2 mg/kg) at a dose that has previously been shown to induce reinstatement behavior [20]. The ethanol was diluted in water to a concentration of 20% *v*/*v*.

### 2.4. Surgery

When required, the rats were implanted with guide cannulas extending to the CeA prior to behavioral training. Prior to intracranial surgery, the rats were injected with the antibiotic Cefazolin (0.03 mL/100 g; i.p.) as an anti-infective antecedent. One 2 mg tablet of the analgesic Rimadyl (bio-serv, Flemington, NJ, USA) was placed on the floor of each subject’s enclosure. The rats were then mounted on a stereotaxic platform following anesthetization with Isoflurane. Cannulae were bilaterally targeted for the CeA (−2.4 AP; ±4.0 ML; −6.9 DV from bregma) and were secured with jewelers’ screws and dental acrylic cement. Post-operative care comprising a Cefazolin injection and a single Rimadyl tablet was administered to the rats for 3 days following the surgical procedures. All the surgical methods were performed using aseptic techniques, and the rats were given 5–7 days of recovery before any experimentation was performed.

### 2.5. Procedure

#### 2.5.1. Ethanol Habituation

Prior to the operant conditioning, all the animals underwent a period of ethanol habituation over a two-week period. During each week, for three consecutive days, animals would receive ethanol (10% *v*/*v*) via standard drinking bottles in their home cages for 10 h. No water was provided at this time. Following ethanol exposure, the animals would receive water ad libitum for two hours, after which they were water-deprived for 12 h. This was repeated for three consecutive days, with the animals receiving water ad libitum for the remaining four days of the week. The bottle and animal weights were recorded before and after ethanol exposure to determine the amount of ethanol consumed. The ethanol consumption across each day of ethanol habituation is presented in Table 1.

#### 2.5.2. Ethanol Self-Administration

The operant conditioning was conducted in daily 1 h sessions, beginning with an FR1 for 7 sessions, then advancing to FR3 for 10 additional sessions or until the criteria were met. The response requirement to increase the FR value was set at a minimum of 15 daily active lever presses for two consecutive days. The criteria for completion of self-administration was set at 35 daily active lever presses for two consecutive days. During the self-administration sessions, a response on the active lever resulted in the opening of the lickometer guillotine door and access to the drinking tube containing the ethanol. The rats were provided access to this drinking tube for 30 s or until they began ethanol (20% *v*/*v*) consumption, whichever came first. Upon initial consumption, they were then given an additional 5 s before the door closed. Pressing of the inactive lever produced no consequence. Animals that did not meet the criteria after 21 sessions (*n* = 4) were removed from the study.

Following the self-administration, the lever press response was extinguished in daily 1 h sessions. During extinction, responding on the active or inactive lever had no scheduled consequence (i.e., no access to drug or stimulus). A minimum of 7 daily sessions and a criteria of less than 20 presses on two consecutive days was required before reinstatement testing, consistent with previous studies employing similar behavioral paradigms [25,26].

**Figure 1:**
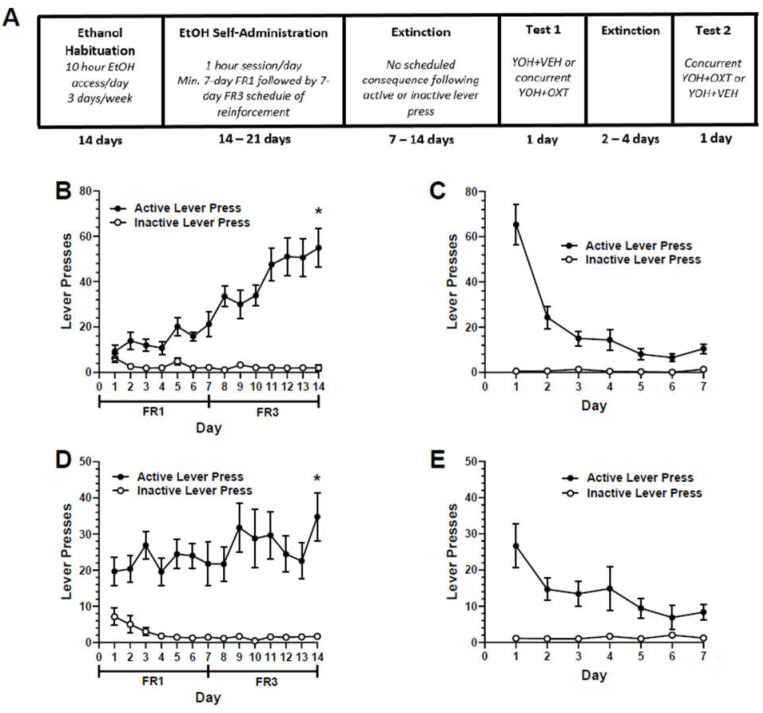
(**A**) Timeline depicting the experimental paradigm across Experiments 1 and 2. All animals underwent ethanol habituation followed by ethanol self-administration, extinction and reinstatement testing. Experiment 1: Active and inactive lever pressing during self-administration across FR1 and FR3 schedules of reinforcement (**B**) and extinction (**C**). Experiment 2: Active and inactive lever pressing during self-administration across FR1 and FR3 schedules of reinforcement (**D**) and extinction (**E**). * denotes significant difference in lever presses relative to Day 1 at *p* < 0.05.

During the stress-induced reinstatement testing, all the animals received injections of the anxiogenic drug, yohimbine (YOH; 2 mg/kg; i.p.), 30 min prior to being placed in the operant chamber. During the reinstatement testing responding on the active or inactive lever continued to have no scheduled consequence. The test sessions lasted 1 h and each rat was tested twice, with at least two extinction sessions occurring between tests, or until the criteria were met. The test conditions were counterbalanced across all the animals. Previous studies have confirmed the absence of a test order effect [27,28,29]. The experimental paradigm and timeline is represented in Figure 1A.

#### 2.5.3. Experiment 1

To determine whether OXT administration was effective at attenuating the YOH-induced reinstatement of ethanol-seeking behavior, all the rats underwent the self-administration, extinction, and reinstatement procedures described above. All the rats (*n* = 11) received two reinstatement tests, one in which they received concurrent injections of OXT (1 mg/kg; i.p.) and YOH (2 mg/kg; i.p.), and another in which they received vehicle (VEH) and YOH. All drug administration occurred 30 min prior to the reinstatement testing. The reinstatement test conditions were randomly counterbalanced across all the rats, with extinction trials between each reinstatement test.

#### 2.5.4. Experiment 2

To establish the role of the CeA in mediating the attenuating effect of OXT on the YOH-induced reinstatement of ethanol-seeking behavior, the rats underwent all the self-administration, extinction, and reinstatement protocols described above. As in Experiment 1, the rats (*n* = 9) received two reinstatement tests, with extinction sessions occurring between each test. Here, the rats received either concurrent intra-CeA infusions of OXT (0.5 µg) and YOH (2 mg/kg; i.p.) or concurrent intra-CeA VEH infusions and YOH (2 mg/kg; i.p.). As with Experiment 1, all the drugs were administered 30 min prior to testing and all the reinstatement test conditions were counterbalanced across all the animals.

### 2.6. Tissue Collection and Histological Analysis

After the second reinstatement test, the rats were decapitated, and their brains were collected for histological assessment of the cannula placement. In brief, the rats were deeply anesthetized with phenytoin/pentobarbital and then transcardially perfused with 150–200 mL cold 0.9% PBS, followed by 200–300 mL of 10% formalin. The brains were removed and post-fixed in 10% formalin for 24 h, submerged in 20% sucrose for 48 h, and then sectioned on a cryostat at 40μm and collected on microscope slides. Tissue slices were stained with Cresyl Violet and a CeA internal cannula placement was confirmed using the Rat Brain in Stereotaxic Coordinates Atlas, 7th edition [30].

### 2.7. Data Analysis

A two-way repeated measures ANOVA was performed to determine the acquisition of operant lever-pressing behavior during self-administration. A one-way ANOVA was used to evaluate the effects of OXT administration on the YOH-induced reinstatement of ethanol-seeking behavior by analyzing the differences between the lever presses between OXT + YOH and VEH + YOH groups, compared to their lever-pressing behavior over the last two days of extinction. Pairwise comparisons were conducted using Dunnett’s multiple comparisons test. All the data were presented as the mean ± S.E.M., and α was set at *p* < 0.05.

## 3. Results

### 3.1. Experiment 1: Systemic OXT Attenuated Yohimbine-Induced Alcohol-Seeking Behavior

**Figure 2:**
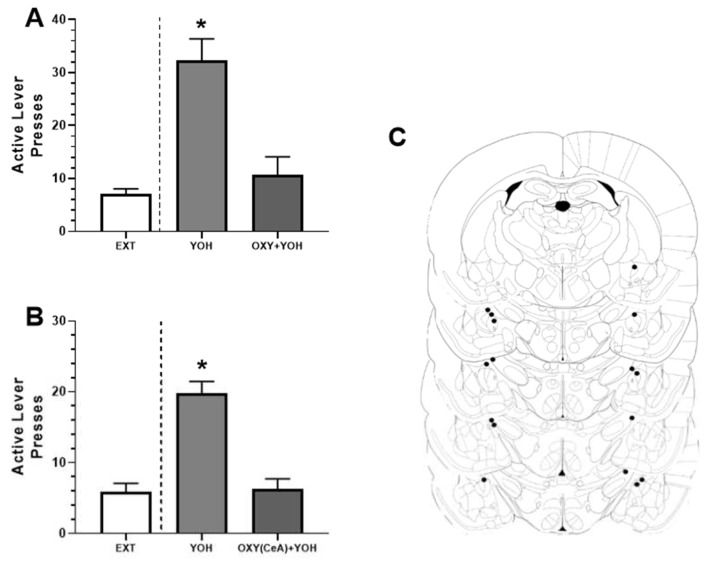
(**A**) Systemic (1 mg/kg) and (**B**) intra-CeA (0.5 µg) administration of OXT attenuated YOH-induced reinstatement of alcohol-seeking. YOH administration results in reinstatement of lever pressing behavior. Concurrent administration of YOH and OXT (systemic or intra-CeA) results in diminished lever pressing behavior. * denotes significant difference in active lever presses relative to EXT (avg. lever presses across last 2 days of extinction) at *p* < 0.05. (**C**) Anatomical depiction of terminal point of the injectors used to infuse OXT into the CeA. EXT = extinction; YOH = yohimbine; OXY = oxytocin; CeA = central amygdala.

All the rats readily pressed the active lever for ethanol and increased their responses over the training period (Figure 1B). A two-way repeated measures ANOVA revealed a significant main effect of training days (F(13, 260) = 10.98, *p* < 0.01), and a post-hoc Dunnett’s multiple comparisons test determined that the rats showed a significant increase in active lever presses on the last day of training relative to their first day (*p* < 0.01). There was no significant difference in inactive lever pressing across all the days. When comparing the lever-pressing behavior of all the animals receiving YOH or YOH + OXT relative to the last two days of extinction (EXT), a one-way ANOVA revealed a significant main effect treatment (F(2, 26) = 20.83, *p* < 0.01). Post-hoc Dunnett’s multiple comparisons tests determined that there was a significant difference in lever presses in the animals that received YOH (M = 32.27) relative to EXT (M = 7.05; *p* < 0.01). There was no significant difference in lever presses in the animals that received YOH + OXT (M = 10.71) relative to EXT (M = 7.05; n.s.). These results suggest that YOH administration successfully reinstates ethanol-seeking behavior and that this effect of YOH is attenuated by the systemic administration of OXT (Figure 2A).

### 3.2. Experiment 2: Intra-CeA OXT Attenuated Yohimbine-Induced Alcohol-Seeking Behavior

All the rats readily pressed the active lever for ethanol across all the training days (Figure 1D). A two-way repeated measures ANOVA did not reveal a significant main effect of the training day (F(13, 208) = 1.52, n.s.), although there was a significant main effect of the lever (F(1, 16) = 52.60, *p* < 0.01), suggesting that the rats consistently pressed the active lever more than the inactive lever. A post-hoc Dunnett’s multiple comparisons test determined that the rats did display a significant increase in active lever presses on the last day of training relative to their first day (*p* < 0.05). There was no significant difference in inactive lever pressing across all the days. When comparing the lever-pressing behavior of all the animals receiving YOH or YOH + OXT (intra-CeA) relative to the last two days of extinction (EXT), a one-way ANOVA revealed a significant main effect of treatment (F(2, 21) = 24.41, *p* < 0.01). The post-hoc Dunnett’s multiple comparisons tests determined that there was a significant difference in lever presses between the animals that received YOH (M = 20.13) relative to EXT (M = 6.38; *p* < 0.01). There was no significant difference in lever presses between the animals that received YOH + OXT (M = 6.25) relative to EXT (M = 6.38; n.s.). These results suggest that, consistent with Experiment 1, YOH administration successfully reinstates ethanol-seeking behavior and that this effect of YOH is attenuated by the intra-CeA administration of OXT (Figure 2B). One animal was excluded from the study due to improper cannula implantation following histological analysis. Figure 2C depicts the terminal point of the injectors used to infuse the compounds into the CeA.

## 4. Discussion

The present study provides evidence that oxytocin, administered both systemically or directly into the CeA, attenuates yohimbine-induced alcohol-seeking behavior. These results provide further support for the interaction between OXT and anxiety-related behaviors. In this study, specifically, we identify that yohimbine effectively reinstated alcohol-seeking behavior as evidenced by an increase in active lever pressing following yohimbine administration. Yohimbine, concurrently administered with OXT, both peripherally and centrally, decreased lever responses, suggesting that OXT directly attenuates the effects of yohimbine on lever pressing behavior.

Most studies investigating the effect of OXT on addiction-related behaviors have often focused on OXT’s direct modulatory effect on the cue- or drug-primed reinstatement of drug-seeking behavior [25,26,31,32,33] (for review see 33) or OXT’s effect on other reward-associated conditioned place preferences [34,35]. A small number of other studies has demonstrated that OXT attenuates stress-induced methamphetamine-conditioned place preference reinstatement [13,36]. Here, we continue to provide evidence that OXT successfully attenuates stress-induced alcohol-seeking behavior. Our results are consistent with previous studies, showing that systemic administration of OXT (at the same dose used in this study; 1 mg/kg) successfully attenuates predator odor-induced and yohimbine-induced alcohol-seeking behavior in mice [37]. Here, we extend these findings to demonstrate that the CeA is an important structure mediating the effect of OXT on stress-induced alcohol-seeking behavior.

Previous studies have demonstrated that OXT acts as a potentially viable anxiolytic neuropeptide. For example, the administration of OXT suppressed stress-related behaviors following situations of social stress [38] and environmental threats [39] in rats. Similarly, the administration of OXT decreased stress-induced corticosterone release [10] and decreased the expression of anxiety-related behaviors in rodents [5,10,11,40] and humans [3]. The OXT-mediated attenuation of yohimbine-induced alcohol-seeking behavior presented here is likely to have been a consequence of the anxiolytic effect of OXT.

While this study did not directly examine the mechanism through which OXT exerts its effect within the CeA, previous studies have implicated the CeA as an important structure in mediating the stress-induced reinstatement of drug-seeking behavior [18,19,20]. Additionally, numerous studies have consistently shown that the CeA expresses high densities of OXT receptors (OXTRs) [15,41]. Specifically, GABAergic interneurons within the CeA express OXTRs. OXTRs are primarily excitatory Gq-coupled metabotropic receptors [41]; these OXTRs are primarily found on PKCδ-expressing GABAergic interneurons [42,43], which play a key role in the inhibition of GABAergic output of the CeA. These OXTR-expressing CeA neurons have been shown to play a critical role in mediating OXT’s anxiolytic effect. OXT acts within the CeA to diminish freezing responses [44] and stress-related behaviors [45]. Hypothalamic OXT neurons project to CeA neuronal populations to exert an OXTR-mediated anxiolytic effect [17] and it has also been established that these OXTR-expressing neurons influenced signaling between the CeA and the periaqueductal gray (PAG) to modulate fear expression [16]. Therefore, it stands to reason that OXT may attenuate yohimbine-induced alcohol-seeking behavior by binding on OXTRs located on these inhibitory GABAergic interneurons in the CeA to attenuate the effect of yohimbine on the reinstatement of alcohol-seeking behavior.

One possible consideration that should be taken into account is whether systemically administered OXT produces this attenuating effect on stress-induced alcohol reinstatement through central mechanisms. The present study demonstrates similar effects of systemic and intra-CeA OXT administration on stress-induced reinstatement of alcohol-seeking behavior, suggesting that systemic OXT may influence signaling in the CeA, either directly or indirectly. Previous studies have shown that the systemic administration of OXT results in the excitation of oxytocin neuronal subpopulations in the paraventricular nucleus (PVN) [29,46] and PVN OXT neurons project directly to the CeA [17], which may provide a mechanism through which systemic OXT may exert an influence on the CeA. Similar studies have demonstrated that peripherally administered OXT results in an increase in central OXT levels in rats [47], while intravenous OXT administration increased OXT levels in the cerebral spinal fluid in rhesus macaques [48].

While the present study highlights that OXT attenuates the stress-induced reinstatement of alcohol-seeking behavior via mechanisms within the CeA, a possible limitation is that it is not immediately clear whether OXT exerts this effect through interaction with OXTRs. Previous studies have demonstrated that OXT may also bind to vasopressin receptors (VPRs). The CeA has previously been shown to express VPRs on GABA projection neurons within the central medial region of the CeA [49,50]. However, VPRs on these subpopulations of GABA projections neurons of the central medial amygdala have previously been shown to drive anxiogenic responses to vasopressin [49] and promote fear expression [50], which is inconsistent with the behavioral effects of OXT shown in the present study, which were more likely to have been driven by the mediation of OXT’s anxiolytic effect by OXTRs [16]. Future studies should elucidate the effect of OXT through the relative contribution of CeA OXTRs and VPRs within the context of stress-induced alcohol-seeking reinstatement. In summary, we reveal several important characteristics related to OXTs’ interaction with stress-induced reinstatement of alcohol-seeking behavior. First, the administration of OXT sufficiently attenuated stress-induced alcohol-seeking behavior in male rats, which highlights a potential pharmacological target to diminish the effect of stress on addiction-related behaviors. Furthermore, we demonstrate that OXT infused directly into the CeA also attenuates the stress-induced reinstatement of alcohol-seeking, which highlights the CeA as a critical structure driving OXT’s effects.

## Figures and Tables

**Table 1:** Means and Standard Deviations for Ethanol Consumption during Ethanol Habituation.

Ethanol Consumption (g/kg)	Experiment 1		Experiment 2	
	M	SD	M	SD
Day 1	2.85	1.67	4.73	1.49
Day 2	4.17	1.87	5.70	1.96
Day 3	3.79	1.88	5.71	2.11
Day 4	5.32	1.45	5.13	0.84
Day 5	6.00	2.10	6.09	0.94
Day 6	6.02	2.20	6.31	1.05

Note. EtoH consumption recorded over 10 h sessions each day. All animals received 3 consecutive days of EtOH habituation per week over two weeks.

## Data Availability

The data presented in this study are available in the present article and on request from the corresponding author.
